# Classification of masked image data

**DOI:** 10.1371/journal.pone.0254181

**Published:** 2021-07-06

**Authors:** Kamila Lis, Mateusz Koryciński, Konrad A. Ciecierski

**Affiliations:** Bioinformatics and Machine Recognition Department, Research and Academic Computer Network, Warsaw, Poland; Sam Houston State University, UNITED STATES

## Abstract

Data classification is one of the most commonly used applications of machine learning. The are many developed algorithms that can work in various environments and for different data distributions that perform this task with excellence. Classification algorithms, just like other machine learning algorithms have one thing in common: in order to operate on data, they must see the data. In the present world, where concerns about privacy, GDPR (General Data Protection Regulation), business confidentiality and security are growing bigger and bigger; this requirement to work directly on the original data might become, in some situations, a burden. In this paper, an approach to the classification of images that cannot be directly accessed during training has been made. It has been shown that one can train a deep neural network to create such a representation of the original data that i) without additional information, the original data cannot be restored, and ii) that this representation—called a masked form—can still be used for classification purposes. Moreover, it has been shown that classification of the masked data can be done using both classical and neural network-based classifiers.

## Introduction

In the present world, the task of classification is common. In almost every aspect of our lives, the classification of our data becomes more useful and essential. To facilitate the machine learning algorithms’ power and get an in-depth analysis of our data, we have to provide the data, perform the analysis, and interpret the results.

The machine learning sophisticated classification algorithms find in–data hidden features and then use them for discrimination. For this, the training data must be provided so that a model describing data can be constructed during the learning process [1].

Furthermore, while this might seem to be an obvious case, more and more often, such a requirement alone proves to be an obstacle. For various reasons, many companies that want to outsource data classification cannot provide virtually any training data. Such a situation can be caused by many reasons, such as corporate policy, security, GDPR, government regulations, legal constraints, etc.

One is faced with a dilemma of how to analyze the data without having direct access to it. The classical approach to data protection, i.e., encryption, is specifically designed to prevent that. The encryption techniques are purposefully designed in such a way that statistical analysis of the encoded form provides no clues about the original data [2].

Another approach is a blind one, i.e., to construct a classifier using the synthetic or publicly available data only, let the customer test it on his own and wait for the results to improve the classifier. Such a process being by nature iterative, is slow and due to constrained feedback of information leads to sub–optimal results.

What is needed is a mapping that, given the original data, would produce its masked form. The masked form would retain the necessary information from the original data, but in a form that is not interpretable by human and, without the inverse mapping, could not be used to reconstruct the original data. Such approach would allow to analyse the data that otherwise could not have been disclosed. Industries that could benefit from such a technique might include medicine, pharmaceutics, and sectors dealing with sensitive or confidential data. There may be many other possible applications as more and more businesses are aware of how much valuable knowledge might be mined from their data.

In this paper, such mapping and its inverse form are proposed using the adversarial autoencoder networks [3] with their latent layers having enforced distributions [4].

Encoded in enforced distributions, latent layers of an adversarial autoencoder can retain information needed for classification, while the output of the network remains as close to the input as desired.

To ensure that original data cannot be easily restored from its latent form, apart from the enforcement of the distribution, the size of the latent layer should be significantly smaller than the size of the input. In this way, the information in the latent layer not only has enforced distributions but it is also compressed in a lossy manner (the autoencoder’s output, while being desirably close to the input, does not have to be equal to it).

## Methodology

In this section, we formalize proposed data masking method and present in detail neural network architectures that have been used.

### Data masking

To address the problem of keeping the data confidential, we propose a solution that uses two neural networks: an adversarial autoencoder and a classifier. First, we trained the adversarial autoencoder on the public image dataset. Then we used the encoder to generate datasets of masked images taken from other public datasets, together with corresponding labels. The masked dataset was then used to train another network to classify the masked representations of images. It has also been shown that for datasets of low complexity, the masked data classification can be done using classical, i.e., non–neural approach.

In our implementation of an adversarial autoencoder, we used two vectors for the latent layer, first, with Gaussian distribution of the data and the second one with a categorical one. These two vectors together form the masked version of the image presented at the input of the encoder.

The Gaussian and categorical distributions were purposefully selected for the latent vectors to facilitate the subsequent classification process. In this way, it is possible to partition the data in an unsupervised way using the categorical part of the latent space and organize each of these partitions internally in a Gaussian way. While this partitioning is fully unsupervised, it still organizes the elements according to their features.

The Gaussian distribution of the latent layer can be obtained using the standard variational autoencoder (VAE) [5]. Still, the use of the adversarial autoencoder allows for the latent layer to be composed of many parts, each with different distribution.

Adversarial autoencoder also produces a better representation of the manifold of the original data in the Gaussian space. In VAE, the Gaussian space contains empty areas that make classification harder as one does not know beforehand what kind of data might in future tasks reside within them [3].

**Fig 1:**
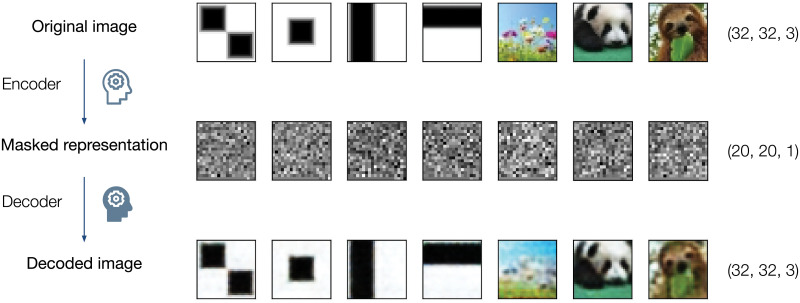
Examples of the masked representation of the input images. Top row represents original images used for the test. Middle row shows Gaussian part of the latent space, reshaped to the square image of the size 20x20. Bottom row represents the output from the decoder.

Forcing the encoder to output latent vectors with Gaussian and categorical distributions [6] makes masked representation noise–like to human eyes while retaining information usable by the neural network–based or classical classifier (see Fig 1). In our research, we prove that the encoder can be used as universal data compression and masking tool that condenses data irreversibly when no proper decoder is provided while preserving information about the input features. Reconstruction of original data is possible only by running the matching decoder, so the decoder weights fulfill the function akin to the private key [2].

### Implementation details

The system architecture corresponds to the two processing phases: masking and classification. Firstly, the image is preprocessed with the encoder part of the adversarial autoencoder, the only part of the model that needs to be saved for masking purposes. The output consists of vectors with Gaussian and categorical distributions. The concatenation of these two vectors constitutes the input for the classification task.

The experiments performed followed these steps:

1. Creating dataset *A* (image, category label)
2. Unsupervised training of the adversarial autoencoder on training image dataset *A*, to obtain a latent image representation; label data is not being used.
3. Creating dataset *B* (image, category label), disjoint with set *A*.
4. Creating a masked dataset *M*(*B*) using the encoder trained in Step 2 (masked_image, category label).
5. Dividing disjointly the *M*(*B*) dataset into *M*(*B*)_training_, *M*(*B*)_validation_ and *M*(*B*)_test_ sets.
6. Training of a classifier based on a *M*(*B*)_training_ set.
7. Testing the classifier basing on the *M*(*B*)_test_ set.

**Fig 2:**
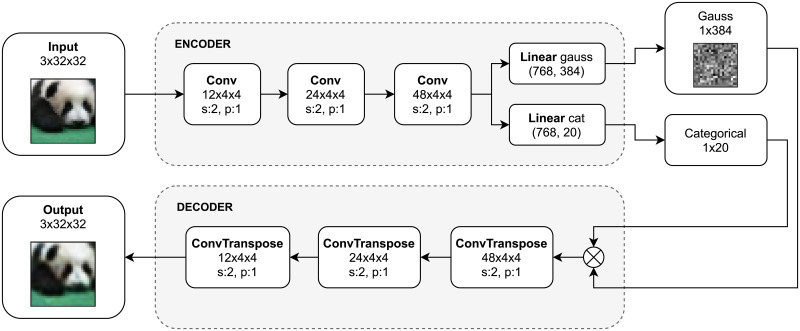
Overview of our encoder-decoder architecture.

The architecture of the autoencoder is presented in Fig 2. Structure of the encoder and decoder parts are shown in S1 and S2 Tables. Discriminators, used in adversarial training, were omitted to simplify the schema, their structure can be found in Supporting information (S3 and S4 Tables). Input image takes masked form in the latent layer that is later an input for the decoder part. The encoder is constructed using three convolutional layers that extract image features and two linear layers, splitting output into the categorical and normal parts. In the input of the decoder, those two vectors are concatenated. The decoder has analogous but transposed convolutional layers as the encoder part.

**Fig 3:**
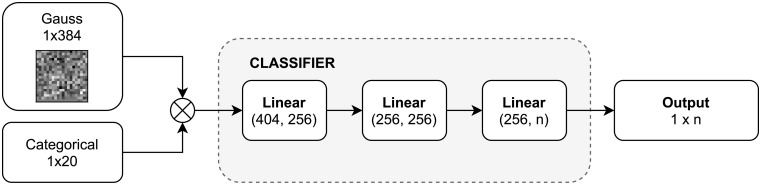
Overview of our masked classifier.

Fig 3 depicts the architecture of the classifier. It takes as input the data produced by the encoder and returns the class of the masked image. The network consists of three linear layers. Two first are activated with the ReLu function, and the last one with Softmax function [4].

**Fig 4:**
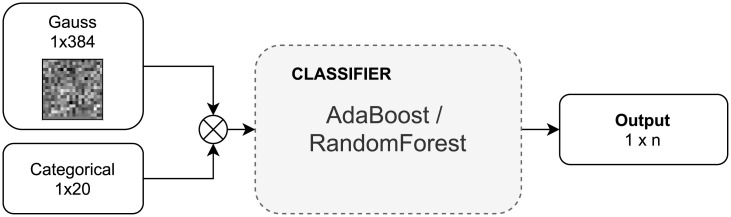
Classification of masked images using Ada Boost and Random Forest classifiers.

We also compared the performance of masked image classification of that neural network with classical machine learning algorithms (see Fig 4). We choose the Random Forest [7] and AdaBoost [8] as they are top–performing classical multi–class classifiers [9].

### Methods

All programming work was done using Python programming language [10]. Autoencoder and neural network classifier were implemented using PyTorch [11] and trained with the help of PyTorch-Ignite [12]. CIFAR and MNIST datasets were managed using PyTorch data loaders. For training classical classifiers, AdaBoost [8] and Random Forest [7], we used scikit-learn library [13]. Mathematical work, as well as matrix and vector manipulations, were done using Numpy [14]. For creation of figures we used Matplotlib [15], Pillow [16], and Draw.io. Plots presenting neural network training sessions were obtained with Tensorboard, part of Tensorflow library [17].

## Experiments

In this section, we analyze the performance of our data masking method for two classification tasks experimentally. We present a description of used datasets and metrics, introduce a generic adversarial autoencoder baseline and show the ability to classify encoded data.

### Datasets and evaluation metrics

In this study, we used three public datasets. We trained autoencoder with CIFAR-10 [18], which contains 60000 images grouped in 10 classes (6000 each). Labels, which were not used in adversarial training, were omitted.

#### Fist classification task

The classifier was tested on masked images from CIFAR-100 [18] which contains 60000 images divided into 100 classes (600 each), and on MNIST [19] dataset containing 60000 training images and 10000 test images, divided into ten classes.

In the case of CIFAR-100 [18] we tested the classification of masked images taken from 2, 3, and 4 randomly selected classes. To avoid biased results, for each category, the choice of classes was made ten times, and obtained results were averaged. Tests on CIFAR-100 [18] dataset were thus performed 30 times in total, ten times using two randomly selected classes, ten times using three randomly selected classes and finally, ten times using four randomly selected classes.

#### Second classification task

The MNIST case was used to show that for input data of low complexity, the classification of masked data can be successfully performed using classical, i.e., non-neural approach. Images from the MNIST dataset were transformed to RGB and resized to 32x32x3—i.e., the size of images in the CIFAR dataset.

CIFAR-100 is divided into train and test sets. There are 50k training images and 10k test images. We extracted the first 10k samples from a training set for validation purposes during the training of the classifier (see [4]). For classical machine learning approaches, there is no need to extract a validation set from a training set. Thus, we merged training and test sets to apply stratified split, resulting in a training to test images ratio of 8:2.

### Adversarial training

Training adversarial autoencoder consists of two phases called reconstruction and regularization [3].

**Fig 5:**
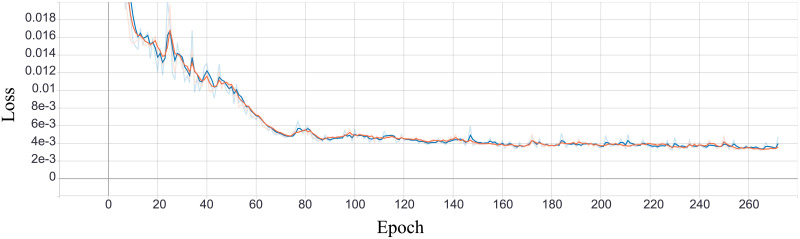
Reconstruction loss.

The first phase is the same as training simple autoencoder—encoder and decoder learn to reconstruct input image into the output image. The similarity of images is evaluated with the mean squared error (squared L2 norm) [4]. During training, the reconstruction error should decrease until it reaches the desired value (see Fig 5).

**Fig 6:**
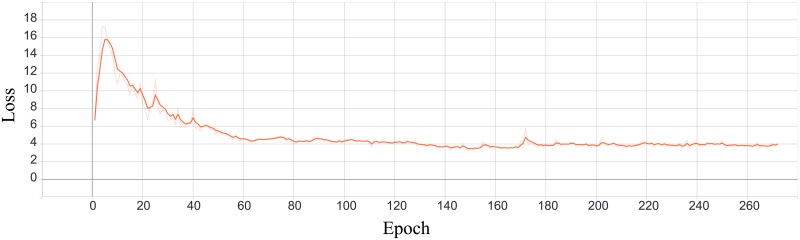
Generating loss.

**Fig 7:**
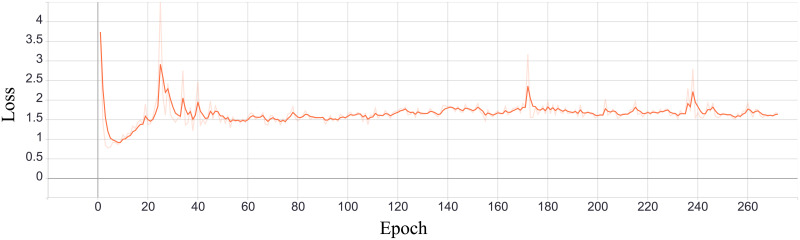
Discriminators total loss (sum of categorical discriminator’s and normal discriminator’s loss values during training).

The second phase aims to shape the latent vector so that information has the given distribution. A discriminator learns to classify input, in terms of distribution, as real or fake. It gets random vector with values sampled from desired distribution with label *real* and encoder output labeled as *fake*. Based on discriminator feedback, the encoder trains to generate values with correct distribution, i.e., it is trained to produce output for which the discriminator would return *real*. For regularization purposes, we measured the Binary Cross Entropy [4] between the target and the output. During training, it is expected for generating loss to decrease and for discriminators loss to increase (encoder becomes so good that discriminator cannot correctly distinguish it from *real* i.e., sampled vector). The process of training those two networks are depicted in Figs 6 and 7.

All models were trained with Adam optimizer [20] with default hyperparameters (learning rate *lr* = 0.001 and coefficients *betas* = (0.9, 0.999)) with a mini-batch size 16. Choosing the size of the latent space is an important problem in autoencoder training. We have used 384–long Gaussian distributed vector and 20–long categorically distributed vector. These values allowed to achieve a satisfactory quality of learning maintaining low consumption of the GPU memory as well as enforced compression of the input data in the latent layer.

### (Encoder, decoder)—The matching pair

During the training of the autoencoder, the encoder and decoder parts are trained together. The training of the network starts with the weights initialized in a random way [21]. Also, during the training, the order of images in batches are randomized [4]. The final weights of the network are thus a product of many processes that, by design, are random in their nature. While it is theoretically possible that two heavily random processes may produce the same result, such a situation is very unlikely. Similarly, it is possible that randomly generated password will unlock access to password–protected data, especially if the password is short or directory–based. Here for the described encoder part of the network, the model has over 400 thousand trainable parameters. To test that each encoder–decoder pair that has been obtained from training is a matched pair, the following experiment has been conducted.

The autoencoder was trained four times using the same training dataset. From training, we have obtained four instances of the autoencoder. Let the *enc*_*j*_ denote encoder transformation from *j*^*th*^ instance of the autoencoder. Let also the *dec*_*j*_ denote decoder transformation from *j*^*th*^ instance of the autoencoder.

**Fig 8:**
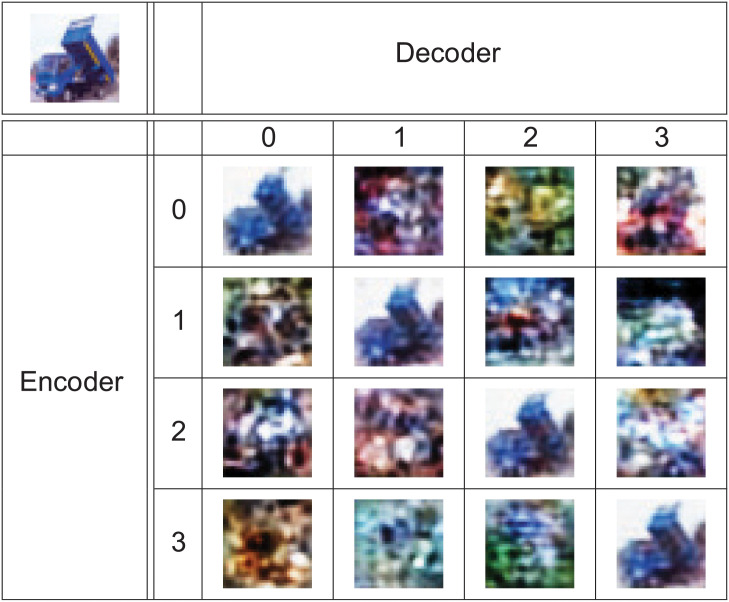
Combinations of (encoder, decoder) pairs from various instances. Results of *dec*_*i*_(*enc*_*j*_(*x*)) where the original image x is shown in upper left corner. The x is properly recreated only if *i* == *j*.

**Fig 9:**
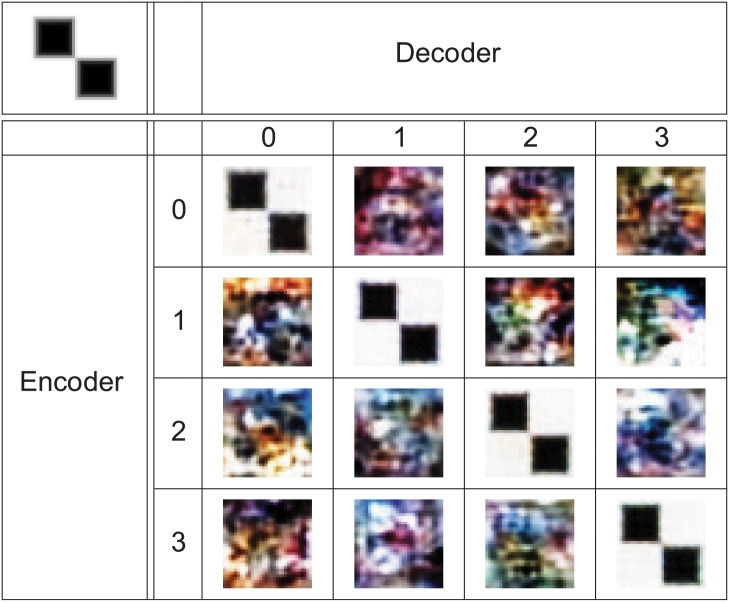
Combinations of (encoder, decoder) pairs from various instances. Results of *dec*_*i*_(*enc*_*j*_(*x*)) where the original image *x* is shown in upper left corner. The *x* is properly recreated only if *i* == *j*. Use of test pattern shows that *dec*_*i*_(*enc*_*j*_(*x*)) for *i* ≠ *j* has no similarity to *x*.

Figs 8 and 9 show that only when encoder and decoder are from the same instance, the output has any similarity to the input.

For set of test patterns *P* (shown in Fig 9 and S1–S3 Figs) the following measures of the MSE [22] were obtained:

$$\begin{array}{c} \mu (MSE{\left(x,dec_{i}\left(enc_{j}\left(x\right)\right)\right)}_{i=j})=0.001811\;\;for\;x\in P \end{array}$$ (1)

$$\begin{array}{c} \sigma (MSE{\left(x,dec_{i}\left(enc_{j}\left(x\right)\right)\right)}_{i=j})=0.000647\;\;for\;x\in P \end{array}$$ (2)

$$\begin{array}{c} \mu (MSE{\left(x,dec_{i}\left(enc_{j}\left(x\right)\right)\right)}_{i\neq j})=0.312014\;\;for\;x\in P \end{array}$$ (3)

$$\begin{array}{c} \sigma (MSE{\left(x,dec_{i}\left(enc_{j}\left(x\right)\right)\right)}_{i\neq j})=0.065740\;\;for\;x\in P \end{array}$$ (4)

For set of 40 000 training images *T* (see Section Datasets and evaluation metrics) the following measures of the MSE were obtained:

$$\begin{array}{c} \mu (MSE{\left(x,dec_{i}\left(enc_{j}\left(x\right)\right)\right)}_{i=j})=0.003543\;\;for\;x\in T \end{array}$$ (5)

$$\begin{array}{c} \sigma (MSE{\left(x,dec_{i}\left(enc_{j}\left(x\right)\right)\right)}_{i=j})=0.000764\;\;for\;x\in T \end{array}$$ (6)

$$\begin{array}{c} \mu (MSE{\left(x,dec_{i}\left(enc_{j}\left(x\right)\right)\right)}_{i\neq j})=0.111649\;\;for\;x\in T \end{array}$$ (7)

$$\begin{array}{c} \sigma (MSE{\left(x,dec_{i}\left(enc_{j}\left(x\right)\right)\right)}_{i\neq j})=0.014910\;\;for\;x\in T \end{array}$$ (8)

For set of 10 000 validating images *V* (see Section Datasets and evaluation metrics) the following measures of the MSE were obtained:

$$\begin{array}{c} \mu (MSE{\left(x,dec_{i}\left(enc_{j}\left(x\right)\right)\right)}_{i=j})=0.003550\;\;for\;x\in V \end{array}$$ (9)

$$\begin{array}{c} \sigma (MSE{\left(x,dec_{i}\left(enc_{j}\left(x\right)\right)\right)}_{i=j})=0.000709\;\;for\;x\in V \end{array}$$ (10)

$$\begin{array}{c} \mu (MSE{\left(x,dec_{i}\left(enc_{j}\left(x\right)\right)\right)}_{i\neq j})=0.112298\;\;for\;x\in V \end{array}$$ (11)

$$\begin{array}{c} \sigma (MSE{\left(x,dec_{i}\left(enc_{j}\left(x\right)\right)\right)}_{i\neq j})=0.012321\;\;for\;x\in V \end{array}$$ (12)

From Eqs 1–12 it is evident that that when encoder and decoder form a matched pair, the mean MSE error is much lower than in the case of unmatched pair. In fact, it is lower by two orders of magnitude. This together with results shown in Figs 8 and 9 and in S1–S6 Figs clearly shows that to decode a data in a masked form, one requires not just any decoder trained with given architecture but the decoder that was trained together with the encoder used for masking process.

### Classification of masked data from the CIFAR-100 dataset

To investigate the information capacity of the masked image data, we performed classification tasks with a neural network classifier consisting of linear layers, as well as classical machine learning algorithms, namely AdaBoost and Random Forest.

In this experiment, the encoder, part of the adversarial autoencoder, was used on the CIFAR-100 dataset images to produce masked representation. Input data for all algorithms consisted of the Gaussian (1*x*384) and Categorical (1*x*20) output of the encoder, joined to create a vector of a size 1*x*404. Classification tasks were performed on all subsets created by subclassing the CIFAR-100 dataset, as described in the previous sections (2–, 3– and 4–class subsets were used).

#### Classification with classical machine learning algorithms

Data were fed into both classifiers, AdaBoost and RandomForest, with original labels associated with images. As for the algorithm, default hyperparameters were used, and models were trained using 100 estimators. After the training step, predictions were made on the test set. Results comprising accuracy, precision and recall are shown in S5–S7 Tables.

#### Classification with neural network

**Fig 10:**
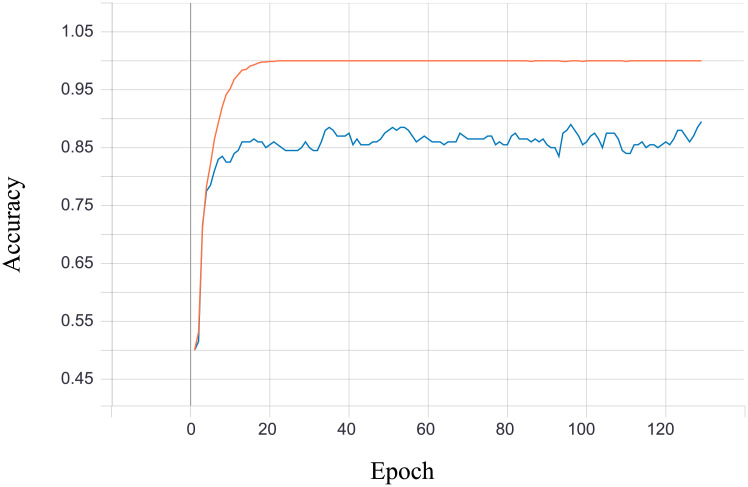
Classification accuracy during training—Case with two classes.

**Fig 11:**
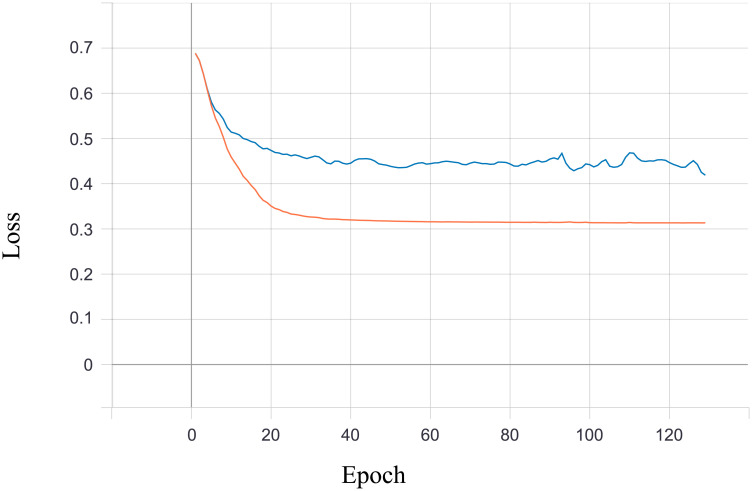
Classification loss during training (2 classes).

The neural network classifier task consists of three linear layers (S8 Table). The activation layers we have chosen are ReLU and Softmax (last layer). The size of the dense layers was chosen arbitrarily based on our experiments with different network configurations. At its input network sees the masked representation of the image, together with the original label in mini-batches of size 4. The model returns, as a prediction, a class an image supposedly belongs to. Model was trained with Adam optimizer with default hyperparameters (learning rate *lr* = 0.001 and *betas* = (0.9, 0.999)) with cross entropy as the loss function. In training, we used a test set disjoint with a validation set. Results calculated for a test set (accuracy, precision and recall) are gathered in S5–S7 Tables. Figs 10 and 11 show accuracy and loss during classifier training in case of two randomly chosen classes. In both plots orange points represents values calculated on the training set, and blue on the validation set.

## Results

Results shown in Tables 1, 2 and S5–S7 Tables indicate that neural network outperforms classical machine learning algorithms in the task of classification of the masked data.

**Table 1:** Classification results for 2, 3 and 4-class subsets drawn from CIFAR100 dataset.

Methods	No classes	Accuracy		Precision		Recall	
		*μ*	*σ*	*μ*	*σ*	*μ*	*σ*
Neural Network	2	0.852	0.068	0.855	0.067	0.851	0.068
	3	0.804	0.090	0.805	0.091	0.804	0.090
	4	0.769	0.098	0.771	0.098	0.769	0.098
Random Forest	2	0.822	0.077	0.822	0.076	0.823	0.077
	3	0.625	0.121	0.748	0.067	0.626	0.119
	4	0.435	0.113	0.746	0.037	0.428	0.102
Ada Boost	2	0.804	0.088	0.805	0.089	0.804	0.088
	3	0.660	0.081	0.670	0.084	0.659	0.082
	4	0.592	0.070	0.600	0.071	0.592	0.070

Results given in this table are averages from ten runs with randomly selected classes. Details for each run are provided in S5–S7 Tables.

**Table 2:** Classification results for MNIST dataset.

Method	Accuracy	Precision	Recall
NN Classifier	0.983	0.983	0.983
Random Forest	0.672	0.920	0.666
Ada Boost	0.689	0.685	0.684

Although results for 2-class cases are comparable (see Table 1 and S5–S7 Tables), the accuracy of Random Forest and AdaBoost classifiers drops much faster, with increasing number of classes, than it is the case with a neural network.

While for 2 classes the accuracies are (0.852, 0.822, and 0.804) for neural network, Random Forest and AdaBoost, respectively, the drop in accuracy when considering four–class experiment is (0.083, 0.387, and 0.212). In the case of classical algorithms (Random Forest and AdaBoost), this drop is by order of magnitude larger than in the case of the neural network–based classifier.

Our tests have shown no significant improvement in the accuracy of the predictions provided by classical algorithms when the number of estimators was increased.

Neural network–based classifier, even with four classes, achieves accuracy close to 80% on masked data.

## Discussion and conclusions

In this study, we propose a novel data masking method using an adversarial autoencoder. Our experiments show it is possible to create a masked form of an image, visually not similar to the original, with retained hidden information sufficient for classification tasks. It has been shown that only the matching decoder is capable of turning masked representation into a form close to the original. This way, we have shown that it is possible to classify the image data without having direct access to it. The owner of the data using the encoding part of the described autoencoder can produce its masked form to provide it for classification. As long as the instance of the autoencoder used for masking remains undisclosed, the classification can be performed without the risk of disclosing the original data. Construction of the autoencoder guarantees that while the information in the latent layer is unreadable to the human, it is still present and can be used for classification and other tasks of choice.

Future work: In future work, an attempt with larger images should be made. While the classification of images from the CIFAR set is proof that such technology can be used, this method should be extended to larger images for many practical approaches. There are many possible ways to approach the extension task, and they should be considered.

Also, the use of autoencoders with the latent layer size in a different ratio to the size of the input, than the one used in this paper, might be considered. A bigger latent layer might provide a better reconstruction of the input, which might provide more informational content for classification. On the other hand, a smaller latent layer would enforce more lossy compression, making the content of the latent layer even more protected from attempts of reverse reconstruction, as well as it would limit the memory demand during the training process.

## Supporting information

S1 File The test patterns. ZIP file containing archived black and white patterns used for autoencoder testing.
(ZIP)

S1 Fig Combinations of (encoder, decoder) pairs from various instances. Results of *dec*_*i*_(*enc*_*j*_(*x*)) where the original image *x* is shown in upper left corner. The *x* is properly recreated only if *i* == *j*. Use of test pattern shows that *dec*_*i*_(*enc*_*j*_(*x*)) for *i* ≠ *j* has no similarity to *x*.
(EPS)

S2 Fig Combinations of (encoder, decoder) pairs from various instances. Results of *dec*_*i*_(*enc*_*j*_(*x*)) where the original image *x* is shown in upper left corner. The *x* is properly recreated only if *i* == *j*. Use of test pattern shows that *dec*_*i*_(*enc*_*j*_(*x*)) for *i* ≠ *j* has no similarity to *x*.
(EPS)

S3 Fig Combinations of (encoder, decoder) pairs from various instances. Results of *dec*_*i*_(*enc*_*j*_(*x*)) where the original image *x* is shown in upper left corner. The *x* is properly recreated only if *i* == *j*. Use of test pattern shows that *dec*_*i*_(*enc*_*j*_(*x*)) for *i* ≠ *j* has no similarity to *x*.
(EPS)

S4 Fig Combinations of (encoder, decoder) pairs from various instances. Results of *dec*_*i*_(*enc*_*j*_(*x*)) where the original image *x* is shown in upper left corner. The *x* is properly recreated only if *i* == *j*.
(EPS)

S5 Fig Combinations of (encoder, decoder) pairs from various instances. Results of *dec*_*i*_(*enc*_*j*_(*x*)) where the original image *x* is shown in upper left corner. The *x* is properly recreated only if *i* == *j*.
(EPS)

S6 Fig Combinations of (encoder, decoder) pairs from various instances. Results of *dec*_*i*_(*enc*_*j*_(*x*)) where the original image *x* is shown in upper left corner. The *x* is properly recreated only if *i* == *j*.
(EPS)

S1 Table Encoder. (PDF)

S2 Table Decoder. (PDF)

S3 Table Normal discriminator. (PDF)

S4 Table Categorical discriminator. (PDF)

S5 Table Classification results for 2-class subsets drawn from CIFAR100 dataset. (PDF)

S6 Table Classification results for 3-class subsets drawn from CIFAR100 dataset. (PDF)

S7 Table Classification results for 4-class subsets drawn from CIFAR100 dataset. (PDF)

S8 Table Masked images classifier. (PDF)

## References

[1] Mitchell TM and others Introduction to machine learning. Machine Learning. 1997;7:2–5. McGraw-hill New York; 1997.

[2] Katz J, Lindell Y Introduction to modern cryptography. CRC press; 2020.

[3] Makhzani A, Shlens J, Jaitly N, Goodfellow I, Frey B Adversarial autoencoders. arXiv preprint arXiv:1511.05644 [Preprint]; 2015. Available from: https://arxiv.org/abs/1511.05644.

[4] Goodfellow I, Bengio Y, Courville A, Bengio Y Deep learning. vol. 1. MIT press Cambridge; 2016.

[5] Kingma DP, Welling M Auto–encoding variational bayes. arXiv preprint arXiv:1312.6114. 2013;. Available from: https://arxiv.org/abs/1312.6114.

[6] Forbes C, Evans M, Hastings N, Peacock B Statistical distributions. John Wiley & Sons; 2011.

[7] Liaw A, Wiener M Classification and Regression by randomForest. R News 2(3):18–22; 2002.

[8] Schapire RE. Explaining adaboost. In: Empirical inference. Springer; 2013. p. 37–52. doi: 10.1007/978-3-642-41136-6_5

[9] Lim TS, Loh WY, Shih YS. A comparison of prediction accuracy, complexity, and training time of thirty-three old and new classification algorithms. Machine learning. 2000; 40(3):203–228. doi: 10.1023/A:1007608224229

[10] Van Rossum G, Drake Jr FL Python tutorial. Centrum voor Wiskunde en Informatica Amsterdam, The Netherlands; 1995.

[11] Paszke A, Gross S, Massa F, Lerer A, Bradbury J, Chanan G, et al. PyTorch: An Imperative Style, High-Performance Deep Learning Library. Advances in Neural Information Processing Systems 32. Curran Associates, Inc.; 2019. p. 8024–8035. Available from: http://papers.neurips.cc/paper/9015-pytorch-an-imperative-style-high-performance-deep-learning-library.pdf.

[12] Fomin V, Anmol J, Desroziers S, Kriss J, Tejani A. High-level library to help with training neural networks in PyTorch; 2020. Available from: https://github.com/pytorch/ignite.

[13] Pedregosa F, Varoquaux G, Gramfort A, Michel V, Thirion B, Grisel O, et al. Scikit-learn: Machine Learning in Python. Journal of Machine Learning Research. 2011; 12:2825–2830.

[14] Harris CR, Millman KJ, van der Walt SJ, Gommers R, Virtanen P, Cournapeau D, et al. Array programming with NumPy. Nature. 2020;585(7825):357–362. doi: 10.1038/s41586-020-2649-2 32939066 PMC7759461

[15] Hunter JD. Matplotlib: A 2D graphics environment. Computing in science & engineering. 2007;9(3):90–95. doi: 10.1109/MCSE.2007.55

[16] Pillow CA (PIL Fork) Documentation; 2015. Available from: https://buildmedia.readthedocs.org/media/pdf/pillow/latest/pillow.pdf.

[17] Abadi M, Agarwal A, Barham P, Brevdo E, Chen Z, Citro C, et al. TensorFlow: Large-Scale Machine Learning on Heterogeneous Systems; 2015. Available from: https://www.tensorflow.org/.

[18] Krizhevsky A Learning Multiple Layers of Features from Tiny Images. 2009.

[19] LeCun Y, Cortes C. MNIST handwritten digit database. 2010.

[20] Kingma DP, Ba J Adam: A method for stochastic optimization. arXiv preprint arXiv:14126980. 2014;. Available from: https://arxiv.org/abs/1412.6980.

[21] Yam JY, Chow TW A weight initialization method for improving training speed in feedforward neural network. Neurocomputing. 2000;30(1-4):219–232. doi: 10.1016/S0925-2312(99)00127-7

[22] Wang Z, Bovik AC Mean squared error: Love it or leave it? A new look at signal fidelity measures. IEEE signal processing magazine. 2009;26(1):98–117. doi: 10.1109/MSP.2008.930649

