# Supplementary figures and images for: Classification of masked image data
Source: PLoS One. 2021 Jul 6;16(7):e0254181. doi: 10.1371/journal.pone.0254181 (PMC8259988; doi:10.1371/journal.pone.0254181)

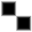

Supplement: S1 File — ZIP file containing archived black and white patterns used for autoencoder testing. (ZIP) [file pone.0254181.s001.zip › supplementary_files/0.png]

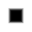

Supplement: S1 File — ZIP file containing archived black and white patterns used for autoencoder testing. (ZIP) [file pone.0254181.s001.zip › supplementary_files/1.png]

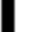

Supplement: S1 File — ZIP file containing archived black and white patterns used for autoencoder testing. (ZIP) [file pone.0254181.s001.zip › supplementary_files/2.png]

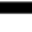

Supplement: S1 File — ZIP file containing archived black and white patterns used for autoencoder testing. (ZIP) [file pone.0254181.s001.zip › supplementary_files/3.png]
