# Supplementary material for: Classification of masked image data
Source: PLoS One. 2021 Jul 6;16(7):e0254181. doi: 10.1371/journal.pone.0254181 (PMC8259988; doi:10.1371/journal.pone.0254181)
Supplement: S1 Table — (PDF) [file pone.0254181.s008.pdf]

S1 **Table. Encoder.**

**Encoder**	Act.	Output shape	Stride	Padding
Input image	–	3x32x32	–	–
Conv 4x4	LeakyReLU	12x16x16	2	1
Conv 4x4	LeakyReLU	24x8x8	2	1
Conv 4x4	LeakyReLU	48x4x4	2	1
Fully-connected	linear	384x1x1	–	–
Fully-connected	linear	20x1x1	–	–
