# Supplementary material for: Classification of masked image data
Source: PLoS One. 2021 Jul 6;16(7):e0254181. doi: 10.1371/journal.pone.0254181 (PMC8259988; doi:10.1371/journal.pone.0254181)
Supplement: S2 Table — (PDF) [file pone.0254181.s009.pdf]

S2 **Table. Decoder**.

**Decoder**	Act.	Output shape	Stride	Padding
Latent vector	–	404x1x1	–	–
ConvTranspose 4x4	LeakyReLU	24x8x8	2	1
ConvTranspose 4x4	LeakyReLU	12x16x16	2	1
ConvTranspose 4x4	LeakyReLU	3x32x32	2	1
