# Supplementary material for: Classification of masked image data
Source: PLoS One. 2021 Jul 6;16(7):e0254181. doi: 10.1371/journal.pone.0254181 (PMC8259988; doi:10.1371/journal.pone.0254181)
Supplement: S3 Table — (PDF) [file pone.0254181.s010.pdf]

S3 **Table. Normal discriminator**.

**Discriminator**	Act.	Output shape
Latent vector	–	384x1x1
Fully-connected	ELU	1000x1x1
Fully-connected	ELU	1000x1x1
Fully-connected	linear	1x1x1
