# Supplementary material for: Classification of masked image data
Source: PLoS One. 2021 Jul 6;16(7):e0254181. doi: 10.1371/journal.pone.0254181 (PMC8259988; doi:10.1371/journal.pone.0254181)
Supplement: S5 Table — (PDF) [file pone.0254181.s012.pdf]

S5 **Table. Classification results for 2-class subsets drawn from CIFAR100 dataset.**

Classes	NeuralNetwork			RandomForest			AdaBoost		
	Acc	Prec	Rec	Acc	Prec	Rec	Acc	Prec	Rec
37, 89	0.775	0.778	0.775	0.650	0.657	0.654	0.642	0.642	0.642
54, 55	0.870	0.874	0.870	0.871	0.870	0.871	0.867	0.867	0.867
14, 44	0.755	0.759	0.755	0.800	0.800	0.800	0.758	0.758	0.758
11, 81	0.795	0.796	0.795	0.779	0.779	0.779	0.762	0.764	0.762
16, 47	0.925	0.926	0.925	0.842	0.841	0.844	0.887	0.889	0.886
46, 95	0.920	0.921	0.920	0.908	0.910	0.910	0.875	0.875	0.875
52, 78	0.960	0.961	0.960	0.925	0.925	0.925	0.946	0.947	0.945
48, 64	0.835	0.843	0.835	0.812	0.812	0.809	0.771	0.772	0.771
55, 95	0.820	0.826	0.820	0.838	0.837	0.838	0.775	0.775	0.774
35, 87	0.860	0.862	0.860	0.792	0.793	0.795	0.758	0.758	0.758
μ	0.852	0.855	0.851	0.822	0.822	0.823	0.804	0.805	0.804
σ	0.068	0.067	0.068	0.077	0.076	0.077	0.088	0.089	0.088
