# Supplementary material for: Classification of masked image data
Source: PLoS One. 2021 Jul 6;16(7):e0254181. doi: 10.1371/journal.pone.0254181 (PMC8259988; doi:10.1371/journal.pone.0254181)
Supplement: S6 Table — (PDF) [file pone.0254181.s013.pdf]

S6 **Table. Classification results for 3-class subsets drawn from CIFAR100 dataset.**

Classes	NeuralNetwork			RandomForest			AdaBoost		
	Acc	Prec	Rec	Acc	Prec	Rec	Acc	Prec	Rec
13, 48, 92	0.797	0.797	0.797	0.528	0.749	0.534	0.661	0.663	0.654
28, 41, 89	0.777	0.776	0.777	0.650	0.762	0.655	0.667	0.669	0.668
2, 18, 87	0.827	0.828	0.827	0.572	0.736	0.568	0.650	0.655	0.652
22, 43, 93	0.707	0.706	0.707	0.628	0.773	0.623	0.603	0.602	0.601
27, 55, 74	0.543	0.542	0.543	0.400	0.640	0.407	0.489	0.491	0.485
18, 65, 68	0.780	0.778	0.780	0.678	0.780	0.676	0.664	0.684	0.664
30, 38, 86	0.817	0.824	0.817	0.842	0.867	0.839	0.792	0.793	0.791
45, 86, 92	0.733	0.733	0.733	0.594	0.662	0.592	0.650	0.667	0.650
27, 37, 52	0.833	0.837	0.833	0.756	0.803	0.759	0.753	0.769	0.752
22, 32, 68	0.743	0.738	0.743	0.603	0.703	0.608	0.675	0.707	0.675
μ	0.804	0.805	0.804	0.625	0.748	0.626	0.660	0.670	0.659
σ	0.090	0.091	0.090	0.121	0.067	0.119	0.081	0.084	0.082
