# Supplementary material for: Classification of masked image data
Source: PLoS One. 2021 Jul 6;16(7):e0254181. doi: 10.1371/journal.pone.0254181 (PMC8259988; doi:10.1371/journal.pone.0254181)
Supplement: S7 Table — (PDF) [file pone.0254181.s014.pdf]

S7 **Table Classification results for 4-class subsets drawn from CIFAR100 dataset.**

Classes	NeuralNetwork			RandomForest			AdaBoost		
	Acc	Prec	Rec	Acc	Prec	Rec	Acc	Prec	Rec
10, 16, 31, 89	0.638	0.645	0.638	0.319	0.742	0.319	0.475	0.475	0.475
38, 66, 72, 89	0.543	0.545	0.543	0.321	0.747	0.331	0.481	0.503	0.482
26, 54, 71, 74	0.740	0.736	0.740	0.429	0.765	0.431	0.629	0.643	0.620
4, 35, 59, 88	0.665	0.668	0.665	0.402	0.783	0.387	0.569	0.578	0.573
10, 59, 64, 89	0.665	0.669	0.665	0.375	0.761	0.379	0.577	0.578	0.579
23, 41, 54, 92	0.790	0.792	0.790	0.571	0.705	0.570	0.656	0.683	0.660
20, 30, 69, 78	0.750	0.750	0.750	0.540	0.730	0.517	0.667	0.664	0.667
1, 3, 42, 60	0.788	0.789	0.788	0.610	0.814	0.576	0.656	0.673	0.666
20, 27, 28, 58	0.743	0.745	0.743	0.487	0.725	0.466	0.571	0.572	0.576
60, 66, 74, 81	0.670	0.673	0.670	0.294	0.690	0.301	0.635	0.629	0.625
μ	0.769	0.771	0.769	0.435	0.746	0.428	0.592	0.600	0.592
σ	0.098	0.098	0.098	0.113	0.037	0.102	0.070	0.071	0.070
