# Supplementary material for: Classification of masked image data
Source: PLoS One. 2021 Jul 6;16(7):e0254181. doi: 10.1371/journal.pone.0254181 (PMC8259988; doi:10.1371/journal.pone.0254181)
Supplement: S8 Table — (PDF) [file pone.0254181.s015.pdf]

S8 **Table. Masked images classifier.**

**Classifier**	Act.	Output shape	Params
Latent vector	–	384	–
Linear	ReLU	256	
Dropout	–	–	0.3
BatchNorm	–	256	
Linear	ReLU	256	
Dropout	–	–	0.3
BatchNorm	–	256	
Linear	Softmax	n	
